# A case report of splenic injury related to colonoscopy: Fortunately treated with conservative treatment

**DOI:** 10.1002/deo2.287

**Published:** 2023-09-01

**Authors:** Kazuya Moriwake, Hiroshi Isozaki, Takehiro Takama, Shigeki Murakami, Sasau Matsumoto

**Affiliations:** ^1^ Department of Surgery Oomoto Hospital Okayama Japan

**Keywords:** abdominal compression, colonoscopy, conservative treatment, splenic injury, traction

## Abstract

Colonoscopy is a common procedure for screening of colon cancer. Although complications are rare, recently there have been reports of splenic injury associated with colonoscopy. Its causes are not clear. Herein, we report an 84‐year‐old man who underwent a colonoscopy for an annual routine examination. The colonoscopy was performed with moderate difficulty due to loop formation and took about 50 min. After the examination, he developed syncope, sweating, and abdominal distention with low blood pressure. Plain computed tomography revealed ascites, and the patient was hospitalized with close monitoring. The following day, his hemoglobin level was decreased by about 3.0 g/dL. Contrast‐enhanced computed tomography revealed the splenic injury. The patient was hemodynamically stable and was treated conservatively. Splenic injury is an uncommon complication of colonoscopy; however, it may cause hemodynamic instability. Physicians performing colonoscopies should be aware of this potential complication.

## INTRODUCTION

Colonoscopy is a common screening test to detect colon cancer. Although it is generally regarded as a safe examination, complications have been reported, such as bowel perforation and intraluminal bleeding; however, the incidence is quite low. Splenic injury (SI) is also an iatrogenic complication after a colonoscopy that has been reported to have high mortality (up to 5%–10%).^1^, ^2^ The causes of SI remain to be elucidated. However, traction of the splenocolic ligament, direct pressure with the endoscope through splenic flexure, and external abdominal pressure to assist at the insertion of the endoscope have been reported.^3^ Treatments for splenic injury are laparotomy, selective arterial embolization, and conservative treatment. If the patient is hemodynamically unstable, emergent surgery is required. However interventional radiology, such as splenic arterial embolization, may be performed depending on the facility. If the patient's status is stable, conservative treatment is acceptable. Herein, we report a case of SI caused by the insertion maneuver of colonoscopy that was treated conservatively.

## CASE REPORT

**FIGURE 1:**
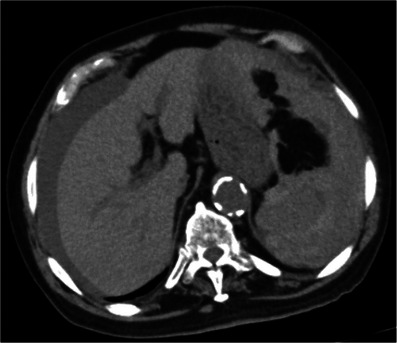
The plain abdominal computed tomography after the colonoscopy. The plain abdominal computed tomography revealed high attenuation ascites but their cause was unclear.

**FIGURE 2:**
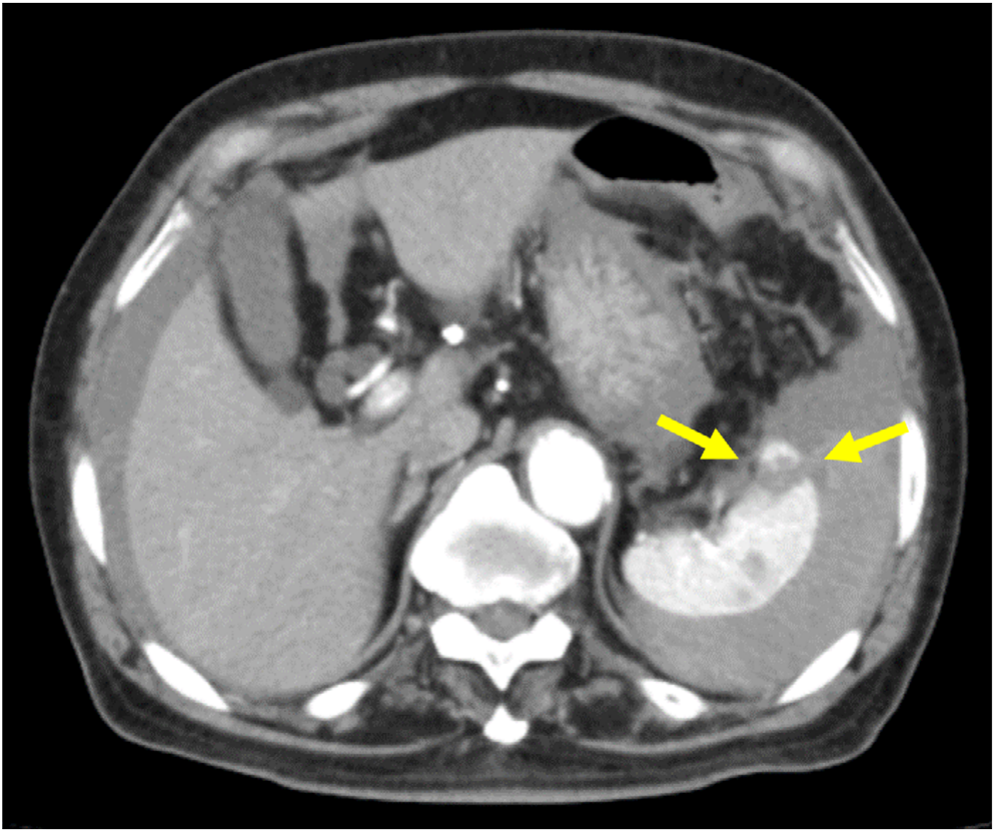
The contrast‐enhanced abdominal computed tomography on the next day. The contrast‐enhanced abdominal computed tomography revealed splenic injury; ascites in the perihepatic, perisplenic, and pelvic area; and low‐density area of the part of the spleen (arrow) without rupture of the splenic artery or extravasation.

An 84‐year‐old man underwent an annual routine colonoscopy. There was moderate difficulty due to loop formation; therefore, the procedure took about 50 min, and a biopsy was performed for a 4 mm polyp at ascending colon. He had hypertension and hyperlipidemia, and a history of cerebral and subarachnoid hemorrhage, percutaneous coronary intervention for angina pectoris, and laparoscopic prostatectomy for prostate cancer. He had been taking an antiplatelet drug due to coronary stents; however, he had not been taking this medication for 1 week during the colonoscopy. About 1 h after the colonoscopy at the recovery unit, the patient developed syncope, sweating, and abdominal distention with a blood pressure of 89/46 mm of mercury (mmHg), respiratory rate of 24 breaths per minute, oxygen saturation of 80% in room air, and heart rate of 59 beats per minute. He was administered intravenous fluids and bed rest. The patient was hemodynamically stable with a blood pressure of 130/82 mmHg, oxygen saturation of 93% on room air, and a heart rate of 68 beats per minute. Abdominal X‐ray showed no free air. The patient had a temporarily decreased level of consciousness and complained of pain in the upper abdomen and left shoulder. Plain computed tomography (CT) from head to pelvis was ordered and revealed high attenuation ascites without intracranial abnormality or abdominal free air (Figure 1). A blood test showed a hemoglobin level of 10.5 g/dL (previous blood test had shown that of 12.3 g/dL), prothrombin time of 11.6 s, and partial thromboplastin time of 26.4 s. Although we diagnosed unexplained intra‐abdominal bleeding, because of the result of the blood test hemoglobin level and coagulation, and the stable vital signs, we selected conservative therapy in our hospital consisting of bed rest, intravenous fluid, hemostatic agent, and clinical and laboratory monitoring. The following day, a blood test revealed a decreased hemoglobin level of 7.7 g/dL. Contrast‐enhanced abdominal CT revealed SI Grade III (classified according to the American Association for the Surgery of Trauma^4^); ascites in perihepatic, perisplenic, and pelvic areas; and low‐density area of the spleen without rupture of splenic artery or extravasation (Figure 2). SI was considered the cause of symptoms and ascites. As the patient didn't complain of increasing pain or develop severe symptoms, conservative treatment was performed with a red blood cell infusion of four units in total. During the 7‐day follow‐up, vital signs have been stable. The patient was discharged with a hemoglobin level of 11.5 g/dL. Two weeks later, abdominal plain CT revealed resolution of ascites and low‐density area of the spleen at outpatient.

## DISCUSSION

Our case developed splenic injury after colonoscopy that was fortunately treated with conservative treatment. However, the diagnosis was delayed because we didn't know this complication.

SI is a relatively rare complication of colonoscopy; however, over 100 case reports of splenic injury after colonoscopy have been reported. Its incidence rate is estimated to be 0.002%–0.033%,^5^, ^6^ which is low compared with other well‐known complications such as bowel perforation (0.035%–0.073%) and intraluminal bleeding (0.065%–0.231%).^6^ The mortality of SI‐related colonoscopy has been reported as 5%–10%.^1^, ^2^ Our case had unpleasant symptoms such as abdominal pain and melena. Physicians performing colonoscopies should be aware of the symptoms associated with this complication.

**FIGURE 3:**
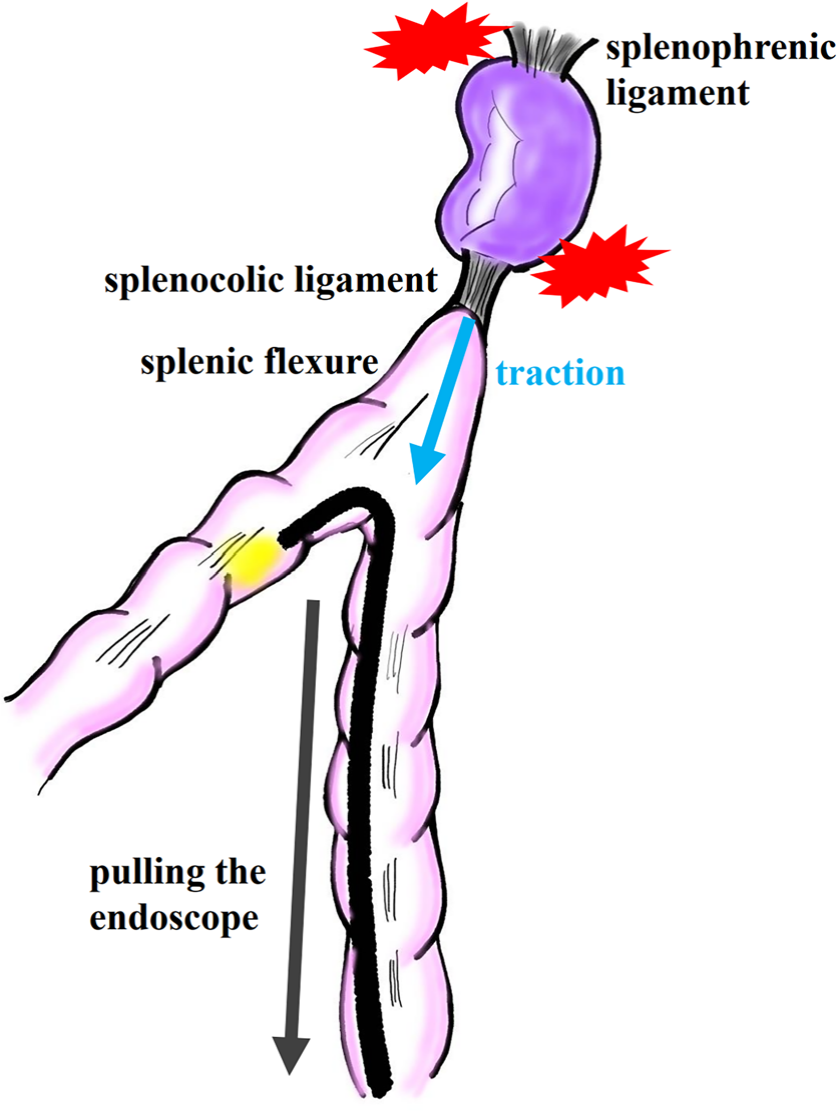
The traction to the ligament of the spleen pulling the colonoscope. The physician angled the scope to release the loop in the sigmoid colon and applied traction with the tip hooked to the splenic flexure.

**FIGURE 4:**
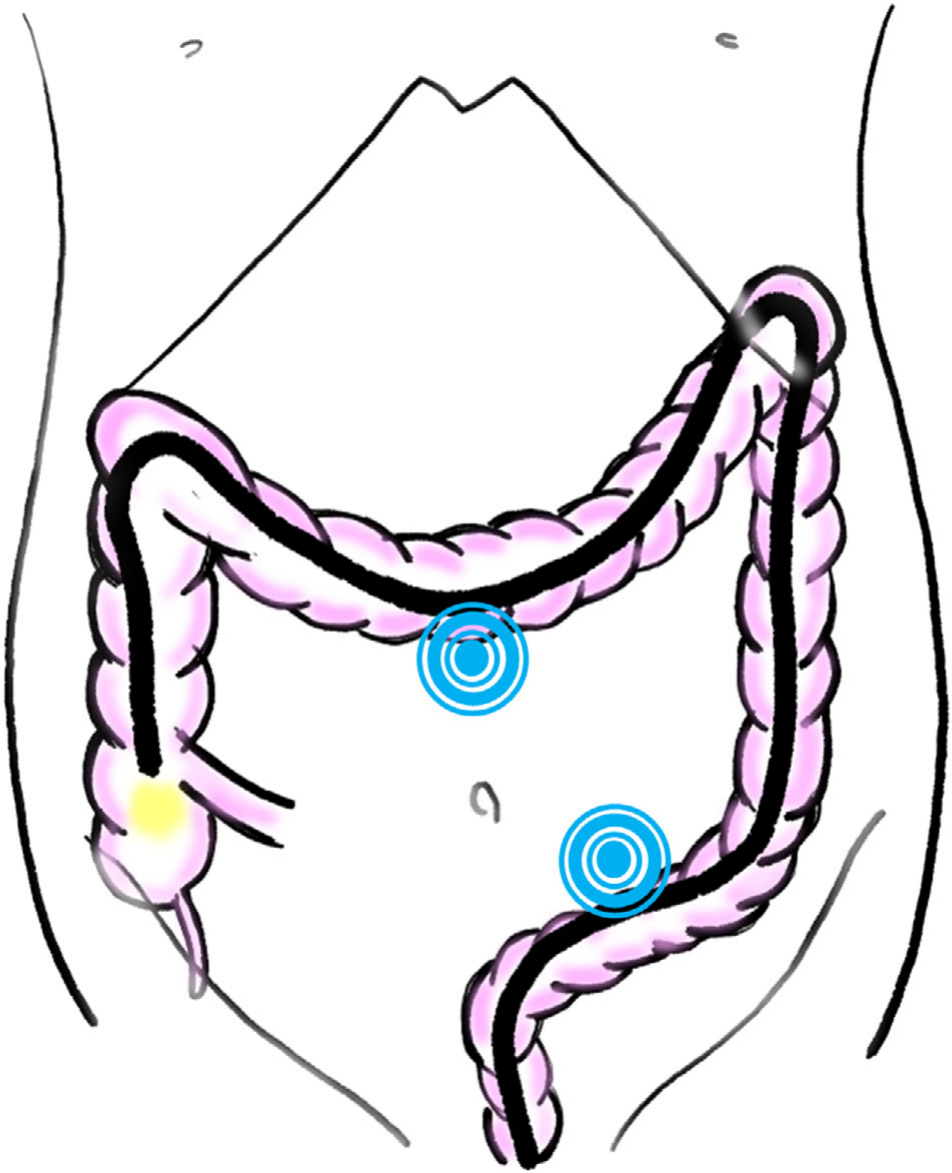
The external abdominal compression to assist the insertion. The assist of manual abdominal compression to supra‐umbilicus and left lower abdomen (two target marks) to prevent stretching of the sigmoid and transverse colon while the insertion by pushing.

The causes of SI in our case were probably external abdominal pressure and endoscopic traction. The insertion of the endoscope was difficult because the sigmoid and transverse colon easily extended and formed loops. Therefore, the physician angled the scope to release the loop in the sigmoid colon and applied traction with the tip hooked to the splenic flexure (Figure 3). Furthermore, when inserting the hepatic flexure, assist of manual pressure was applied to the supra‐umbilicus and left lower abdomen to prevent stretching of the sigmoid and transverse colon while the insertion by push (Figure 4). These maneuvers probably gave a tractional force to the spleen through the splenocolic ligament. In addition, the splenic injury was located on the lower ventral site of the spleen in our case. There are two conceivable mechanisms. First, there was a possibility that tension on the splenocolonic ligament and splenophrenic ligament due to traction from the scope caused the spleen to get cracked on the ventral side simply. Secondly, another conceivable mechanism is as follows: the traction from the scope and strong compression in two abdominal locations caused the spleen to move, leading to a splenic crack due to the adhesions between the spleen and abdominal wall or greater omentum. Although this is purely speculative, adhesions forming around the spleen are relatively common in laparotomy, from the standpoint of a surgeon. The physician should be careful of excessive traction to the splenic flexure and multiple manual compressions in the presence of resistance. Excessive hardening of the scope during push insertion should also be considered with caution. Recent studies reported traction of the splenocolic ligament, direct pressure with the endoscope through splenic flexure, and external abdominal pressure to assist at the insertion of an endoscope as causes.^3^ In addition, the adhesion between the colon and spleen due to previous history of abdominal surgery is considered a cause in other reports: about half of patients had a history of previous abdominal surgery.^2^, ^7^ Our case had undergone pelvic surgery; however, no definitive relationship with adhesion around the spleen was found. Another possible cause was the antiplatelet drug, but it was stopped for one week before the colonoscopy.

Immediate contrast‐enhanced CT was needed to detect and diagnose SI. In our case, although plain CT as a primary examination did not detect SI, we decided to stay on as a follow‐up admission because of hemodynamic stability. However, high attenuation ascites were revealed, so at least contrast‐enhanced CT should be taken to diagnose. According to a recent study, contrast‐enhanced CT diagnosed 98.5% of splenic trauma.^1^ It's also able to reveal the grade of SI and the amount of hemorrhage objectively. Furthermore, focused assessment with ultrasonography for trauma is also helpful to detect hematogenous ascites.^8^ Didactically, immediate contrast‐enhanced CT due to rule out SI as a complication after colonoscopy is necessary along with bowel perforation and intraluminal bleeding.

Since our case had been hemodynamically stable due to bed rest and intravenous fluid, we selected conservative treatment with follow‐up observation; thus, laparotomy was not performed, and the spleen was preserved. The treatment for SI includes emergent splenectomy; interventional radiology, such as splenic arterial embolization; and conservative treatment. Previous studies reported that splenectomy was performed for patients with hemodynamic instability, and it's the most frequent treatment (about 70%) for this complication.^2^, ^7^ Recently, conservative management and splenic artery embolization that preserves splenic function have been increasingly preferred for the treatment of SI.^9^ These non‐operative management for SI has been reported at rates of up to 70%, and its success rate is approximately 70%–90%.^10^ It is considered acceptable for the treatment of patients with hemodynamic stability. Previous studies reported that hemodynamic stability is the most decisive factor to perform operative treatment regardless of the grade of splenic injury and decreased hemoglobin value.^1^, ^2^, ^7^

In conclusion, colonoscopy‐related SI is a rare lethal complication. Physicians should be careful of the excessive traction with the splenic flexure to release the loop in the sigmoid colon or multiple external abdominal compression to assist the insertion of colonoscopy. For diagnosis and deciding the treatment for this complication, immediate contrast‐enhanced CT is necessary.

## CONFLICT OF INTEREST STATEMENT

None.

## INFORMED CONSENT

Informed consent was obtained from the patient for the publication of this case report and any accompanying images.
